# Childhood Obesity and Risk of Stroke: A Mendelian Randomisation Analysis

**DOI:** 10.3389/fgene.2021.727475

**Published:** 2021-11-17

**Authors:** Xue-Lun Zou, Sai Wang, Lei-Yun Wang, Lin-Xiao Xiao, Tian-Xing Yao, Yi Zeng, Le Zhang

**Affiliations:** ^1^ Department of Neurology, Xiangya Hospital, Central South University, Changsha, China; ^2^ Department of Clinical Pharmacology, Hunan Key Laboratory of Pharmacogenetics, Xiangya Hospital, Institute of Clinical Pharmacology, Central South University, Changsha, China; ^3^ Department of Spine Surgery and Orthopaedics, Xiangya Hospital, Central South University, Changsha, China; ^4^ Department of Geriatrics, Second Xiangya Hospital, Central South University, Changsha, China

**Keywords:** childhood obesity, stroke, mendelian randomization, genetics, GWAS

## Abstract

**Background:** The causal relationship between childhood obesity and stroke remains unclear. Our objective was to elucidate the causal relationship between childhood obesity and the risk of stroke and its subtypes by performing Mendelian randomisation (MR) analyses.

**Methods:** Genetic instruments for childhood obesity were obtained from a genome-wide association study (GWAS) of 13,848 European participants. Summary level data for stroke, intracerebral haemorrhage, ischaemic stroke (IS), and its subtypes were evaluated using the MEGASTROKE GWAS dataset, which included 446,696 European adults. Inverse-variance weighting, weighted-median analysis, MR-Egger regression, MR Pleiotropy RESidual Sum and Outlier test (MR-PRESSO), and MR-Robust Adjusted Profile Score were applied in this MR analysis. The leave-one-out sensitivity test, MR-PRESSO Global test, and Cochran’s Q test were conducted to confirm the accuracy and robustness of our results.

**Results:** Genetic evaluations revealed that childhood obesity was associated with a higher risk of stroke (OR = 1.04, 95%CI: 1.01–1.07, *p* = 0.005) and IS (OR = 1.05, 95%CI: 1.02–1.08, *p* = 0.003), but not with intracerebral haemorrhage (ICH, OR = 0.93, 95%CI: 0.80–1.09, *p* = 0.39). In the subtype analysis, childhood obesity was also associated with large artery stroke (LAS, OR = 1.12, 95%CI: 1.02–1.22, *p* = 0.016) but not with cardioembolic stroke (OR = 1.06, 95%CI: 0.96–1.18, *p* = 0.21) and small vessel stroke (OR = 1.06, 95%CI: 0.98–1.15, *p* = 0.17). These results were stable in the sensitivity analysis and remained significant after Bonferroni correction.

**Conclusion:** Our study provides evidence that childhood obesity is associated with a higher risk of stroke, IS, and LAS. The prevention of stroke, especially IS and LAS, should be promoted in populations with childhood obesity.

## Introduction

Stroke is one of the most common cerebrovascular diseases and is the main cause of disability and mortality worldwide (Krishnamurthi et al., 2020). The number of patients with stroke who had a recurrence, died, and survived or remained disabled has increased three-fold (Krishnamurthi et al., 2020). The disease burden of stroke has a great influence on the global economy, especially in low-income countries (Feigin et al., 2016; GBD 2016 Stroke Collaborators, 2019). Traditional risk factors of stroke, such as smoking, alcohol consumption, hypertension, diabetes, and sleep duration, have been applied in prevention and treatment. Some traditional risk factors for childhood obesity have also been discovered.

With economic development and the improvement of elevators in the past 2 decades, the prevalence of obesity in childhood almost doubled in more than 70 developed countries (Ng et al., 2014; de Bont et al., 2020). The highest childhood obesity level has been reported in Americans at 12.7% (Ng et al., 2014; GBD 2015 Obesity Collaborators, 2017). Childhood obesity is easily sustained, increasing the incidence of cardiovascular disease and metabolic syndrome (Weihrauch-Blüher et al., 2019). Observational studies have confirmed that a higher degree of childhood obesity is associated with a higher risk of stroke (Ajala et al., 2017; Gjærde et al., 2017), particularly ischaemic stroke (IS) (Gjærde et al., 2017). However, these studies may be influenced by health and nutritional status and other confounding factors, which may lead to potential reverse relationships.

Mendelian randomisation (MR) is a traditional statistical method used to evaluate the causal relationship between diseases and their risk factors. Compared to conventional epidemiological studies, MR analysis has the power to overcome confounding factors and reverse relationships (Georgakis et al., 2019). It analyses the causal relationship at the genetic level, which treats the instrumental variables as genetic predictors for assessing the causal relationship between exposure and outcome (Davey Smith and Hemani, 2014). Therefore, this study aimed to explore whether childhood obesity is associated with stroke and its subtypes using MR analysis.

## Materials and Methods

### Study Design

**FIGURE 1:**
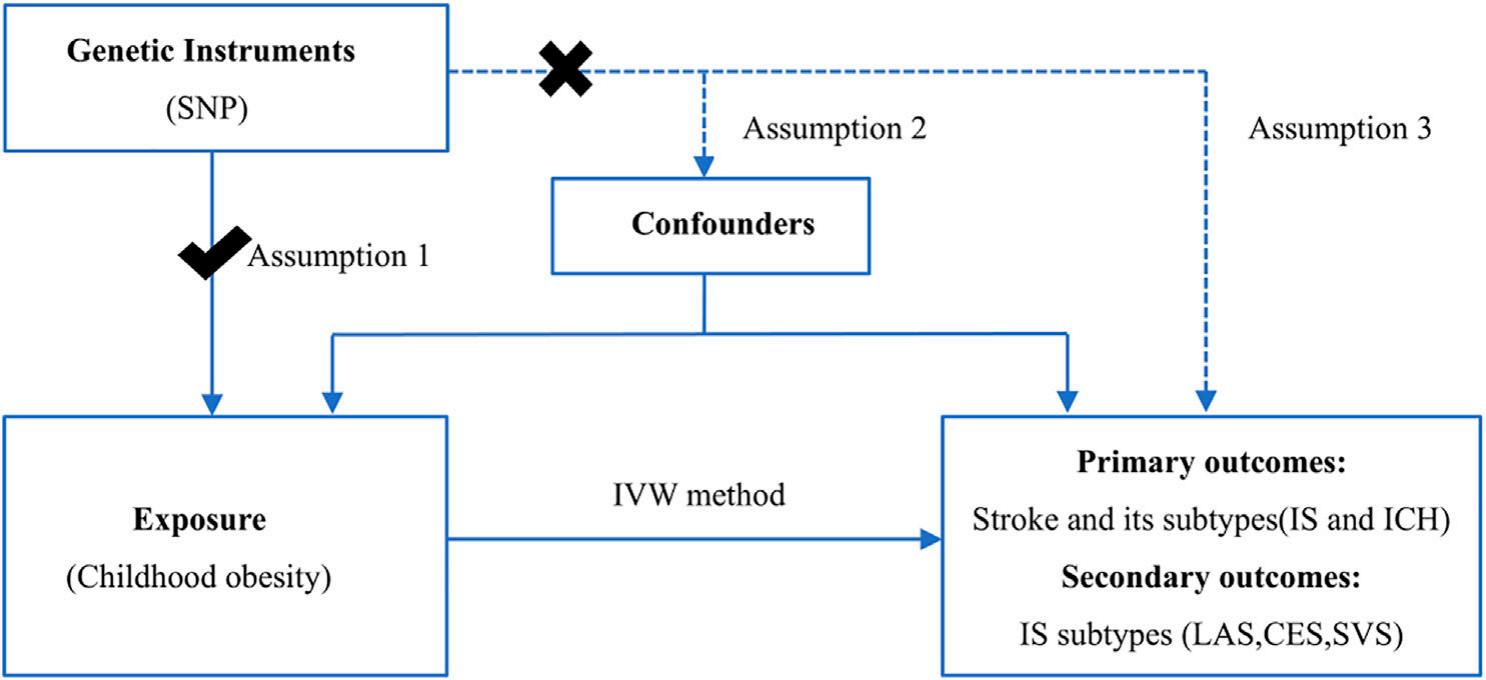
Assumption and design for the Mendelian randomisation study. Three assumptions in this MR study: (Krishnamurthi et al., 2020) Instrumental variables are correlated with exposure; (GBD 2016 Stroke Collaborators, 2019) instrumental variables are not associated with confounding factors; and (Feigin et al., 2016) the instrumental variable is not related to outcome and is only associated with outcome through exposure. IVW, inverse-variance-weighted; SNP, single nucleotide polymorphism; IS, ischemic stroke; LAS, large vessel ischemic stroke; CES, cardioembolic ischemic stroke; SVS, small vessel ischemic stroke; ICH, intracerebral hemorrhage.

As shown in Figure 1, the primary analysis tested whether childhood obesity is associated with stroke and its subtypes [IS and intracerebral haemorrhage (ICH)]. When the causal relationship of IS was confirmed, the secondary analyses tested what subtype of IS, including large artery stroke (LAS), cardioembolic stroke (CES), and small vessel stroke (SVS), has increased by childhood obesity. To correct for false-positive results that may be brought about by the various comparison methods involved in this study, we conducted multiple test corrections (Bonferroni correction). The p-value of this study was two-sided and was defined as <0.017 for a Bonferroni correction of three tests for primary and secondary analyses. The design of this study was based on MR design, and all data were from published studies and public databases, so there was no need for additional ethical review.

### Sources of Genome-Wide Association Study

The genetic instruments of childhood obesity were acquired from a public genome-wide association study (GWAS) dataset which included 5,530 cases and 8,318 controls of European children from the Early Growth Genetics (EGG) consortium (Bradfield et al., 2012). This database is based on a meta-analysis that contained approximately 14 genome-wide association studies and 2.7 million single-nucleotide polymorphisms (SNPs). Childhood obesity was defined as a BMI above 95% in the same age as reported in the EGG consortium (Flegal et al., 2002). Stroke information was obtained from a meta-analysis of 29 GWAS performed by the MEGASTROKE consortium, including approximately 40,585 cases and 406,111 healthy European adults as controls (Malik et al., 2018). In the GWAS meta-analysis, the clinical information on stroke has been described in other studies (Qian et al., 2020). Stroke subtypes were classified as IS (34,217 cases and 406,110 controls) and ICH. IS was divided into LAS (4,373 cases and 146,392 controls), CES (7,193 cases and 204,570 controls), and SVS (5,386 cases and 192,662 controls). Summary level data of ICH were obtained in another GWAS meta-analysis that had European participants with 1,545 cases and 1,481 controls (Woo et al., 2014).

### Selection of SNP in Exposure and Outcome

In an MR study, instrumental variables should be meet three assumptions (Figure 1):

Assumption 1 Instrumental variables are correlated with exposure.

Assumption 2 Instrumental variables are not associated with confounding factors.

Assumption 3 Instrumental variable is not related to outcome, and it is only associated with outcome through exposure.

As shown in Supplementary Table S1, the SNP was significantly related to childhood obesity and that stroke was only influenced by childhood obesity and not by instrumental variables, which is in accordance with Assumptions 1, 3. For Assumption 2, we used phewascatalog (Carroll et al., 2014; Huang and Labrecque, 2019) (phewascatalog.org) to detect whether there is a potential relationship between SNPs and measured or unmeasured confounders. There was no statistically significant relationship between them at a threshold of *p* < 5.0 × 10^–6^, which is consistent with Assumption 2.

SNPs were selected from the GWAS database, as described above. As MR studies require at least 10 instrumental variables (Savage et al., 2018; Gao et al., 2019), only five SNPs can be included in the threshold of *p* < 5 × 10^–8^. We then selected the instrument variants in a more relaxed p-value (*p* < 5 × 10^–6^) to acquire suitable SNPs for this MR analysis (Ference et al., 2015; Gao et al., 2019). The parameters (kb = 10,000 and *r* ^2^ = 0.01) were used to remove the linkage disequilibrium between each variable. For childhood obesity-stroke (including all IS subtypes) MR analysis, the SNP rs1040070 was removed as this was palindromic with intermediate allele frequencies. In addition, two SNPs (rs1040070 and rs9299) were removed because of the lack of available proxies in childhood obesity-ICH MR analysis. In the secondary analysis of childhood obesity with IS subtypes, rs13130484 was excluded because it could not be found in the outcome of CES (Supplementary Tables S1, S2). F statistics were computed to estimate whether a weak instrument bias was present and to improve the power of the selected instrumental variables (Pierce et al., 2011). As shown in Supplementary Tables S1, S2, all F statistics in 15 instrumental SNPs were above the threshold of 10.

### Two-Sample MR Analysis

This two-sample MR analysis was conducted to investigate the potential causal relationship between childhood obesity and the risk of stroke and its subtypes. Through exposure selection, MR analysis was conducted using the inverse-variance-weighted (IVW) model (Burgess et al., 2015), weighted-median estimator (WME) (Bowden et al., 2016), MR-Egger regression method (Bowden et al., 2015), MR Pleiotropy RESidual Sum and Outlier (MR-PRESSO) (Verbanck et al., 2018), and MR-Robust Adjusted Profile Score (Zhao et al., 2020). Among the five methods, the traditional IVW method was used as the main MR analysis to evaluate the causal effect of childhood obesity-stroke because of its stability and accuracy when directional pleiotropy is absent (Burgess et al., 2015). WME can output accurate results when more than 50% of the instrumental variables are invalid (Bowden et al., 2016). Moreover, if horizontal pleiotropy exists, it has the advantage of reducing the type I error and can precisely evaluate causal relationships (Bowden et al., 2016). MR-Egger regression cannot be affected by whether instrumental variables are valid. It can control the base of the directional pleiotropic effect (Bowden et al., 2015). If the instrument strength, independent of the direct effect assumption, is perfected, the MR-RAPS will be submitted as a square of the mean with fewer mistakes (Zhao et al., 2020).

Furthermore, the MR-Egger regression and MR-PRESSO were applied to test for the presence of pleiotropy. If pleiotropy exists, it can be detected using the MR-Egger regression method. MR-PRESSO is a method used to detect the effect of directional pleiotropy. In addition, it can filter potential outliers and assist in correcting them (Bowden et al., 2016). The leave-one-out sensitivity test involves eliminating the SNPs to judge the sensitivity of a single SNP in this MR study (Hemani et al., 2018). The difference between various instrumental variables was analysed by Cochran’s Q test (heterogeneity test).

Similar to meta-analysis, MR analysis can also be analysed by fixed-effect which is the conventional robust selection or random-effect analysis when heterogeneity cannot be addressed. In all sensitivity analyses, the threshold of the p-value was set at 0.05. This two-sample MR analysis used the MR and MR-PRESSO packages in R (version 4.0.3) software.

## Results

### Primary Analysis of Childhood Obesity With the Risk of Stroke and its Subtypes

**FIGURE 2:**
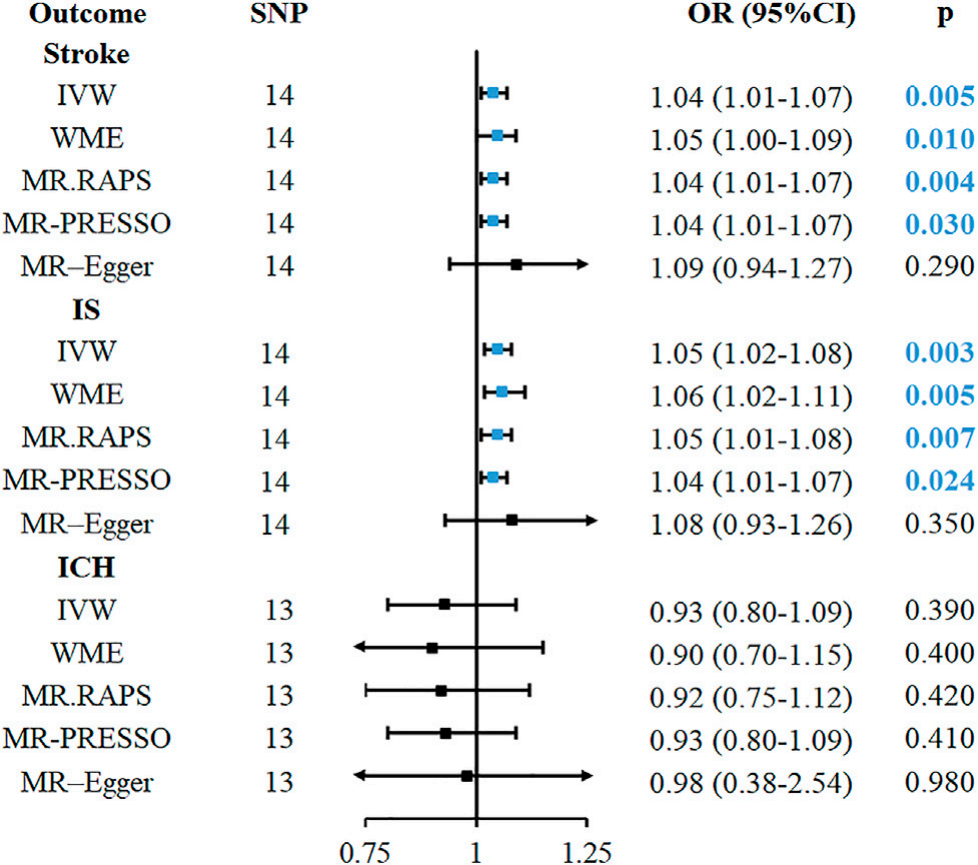
The causal relationship between childhood obesity and risk of stroke and its subtype in Mendelian randomisation. CI, confidence interval; IVW, inverse-variance-weighted; MR, Mendelian randomization; OR, odds ratio; SNP, single nucleotide polymorphism; IS, ischemic stroke; LAS, large vessel ischemic stroke; CES, cardioembolic ischemic stroke; SVS, small vessel ischemic stroke, ICH, intracerebral hemorrhage; MR. RAPS, MR-Robust Adjusted Profile Score; MR-PRESSO, MR pleiotropy residual sum and outlier.

The causal relationship between genetically predicted childhood obesity and the risk of stroke and its subtypes is shown in Figure 2. We found that genetic prediction of childhood obesity was causally associated with the risk of stroke (IVW, OR = 1.04, 95%CI: 1.01–1.07, *p* = 0.005), and can be proven in the WME (OR = 1.05, 95%CI: 1.00–1.09, *p* = 0.01) and MR-RAPS (OR = 1.04, 95%CI: 1.01–1.07, *p* = 0.004). The association between childhood obesity and risk of stroke in the MR analysis was statistically significant (IVW, OR = 1.04, 95%CI: 1.00–1.08, *p* = 0.048) in the genome-wide significance threshold of *p* < 5 × 10^–8^ (Supplementary Table S3). No potential outliers in the selected instrument were found in the MR-PRESSO and leave-one-out sensitivity tests (Supplementary Figure S1A). There was no evidence of heterogeneity (Q = 14.2, *p* = 0.32) in the Cochran’s Q test. The intercept of the MR-Egger regression (*p* = 0.57) and the p-value (*p* = 0.28) of the MR-PRESSO Global test did not show any pleiotropy (Table 1).

**TABLE 1:** Sensitivity analysis of childhood obesity and stroke in MR analysis.

Method	SNP(n)	Cochran’s Q	HT(p)	Intercept	PT(p)	RSSobs	GT(p)
Stroke				-0.0075	0.57	18.9	0.31
MR–Egger	14	13.8	0.32				
IVW method	14	14.2	0.36				
IS				-0.0054	0.69	17.5	0.39
MR–Egger	14	12.5	0.40				
IVW method	14	12.7	0.47				
LAS				-0.0018	0.97	24.6	0.12
MR–Egger	14	20.2	0.06				
IVW method	14	20.2	0.09				
CES				0.0208	0.63	38.3	0.003
MR–Egger	13	30.0	0.003				
IVW method	13	30.7	0.004				
SVS					0.40	28.9	0.04
MR–Egger	14	22.8	0.03	0.0308			
IVW method	14	24.2	0.03				
ICH				−0.0379	0.66	12.2	0.65
MR–Egger	13	8.5	0.96				
IVW method	13	8.5	0.70				

Abbreviation: IVW, inverse-variance-weighted; MR, Mendelian randomization; SNP, single nucleotide polymorphism; HT, heterogeneity test; PT, pleiotropy test; IS, ischemic stroke; LAS, large vessel ischemic stroke; CES, cardioembolic ischemic stroke; SVS, small vessel ischemic stroke; ICH, intracerebral hemorrhage; GT, MR-PRESSO Global Test.

The causal association between childhood obesity and IS was also found in IVW (OR = 1.05, 95%CI: 1.02–1.08, *p* = 0.003), WME (OR = 1.06, 95%CI: 1.02–1.11, *p* = 0.005), and MR-RAPS (OR = 1.05, 95%CI: 1.01–1.08, *p* = 0.007), as shown in Figure 2. MR estimation also had a statistical significance when the p-value was set to less than 5 × 10^–8^ (IVW, OR = 1.05, 95%CI: 1.00–1.09, *p* = 0.04) (Supplementary Table S3). No outliers were detected using the MR-PRESSO outlier test and leave-one-out sensitivity test (Supplementary Figure S1B). The heterogeneity of Cochran’s Q test (Q = 12.7, *p* = 0.47), directional pleiotropy in the MR-Egger regression (*p* = 0.40), and the MR-PRESSO Global test (*p* = 0.39) were not statistically significant. However, the association of childhood obesity with the risk of ICH was not confirmed by MR analysis (IVW, OR = 0.93, 95%CI: 0.80–1.09, *p* = 0.39).

### Secondary Analysis of Childhood Obesity with the Risk of IS and IS Subtypes

**FIGURE 3:**
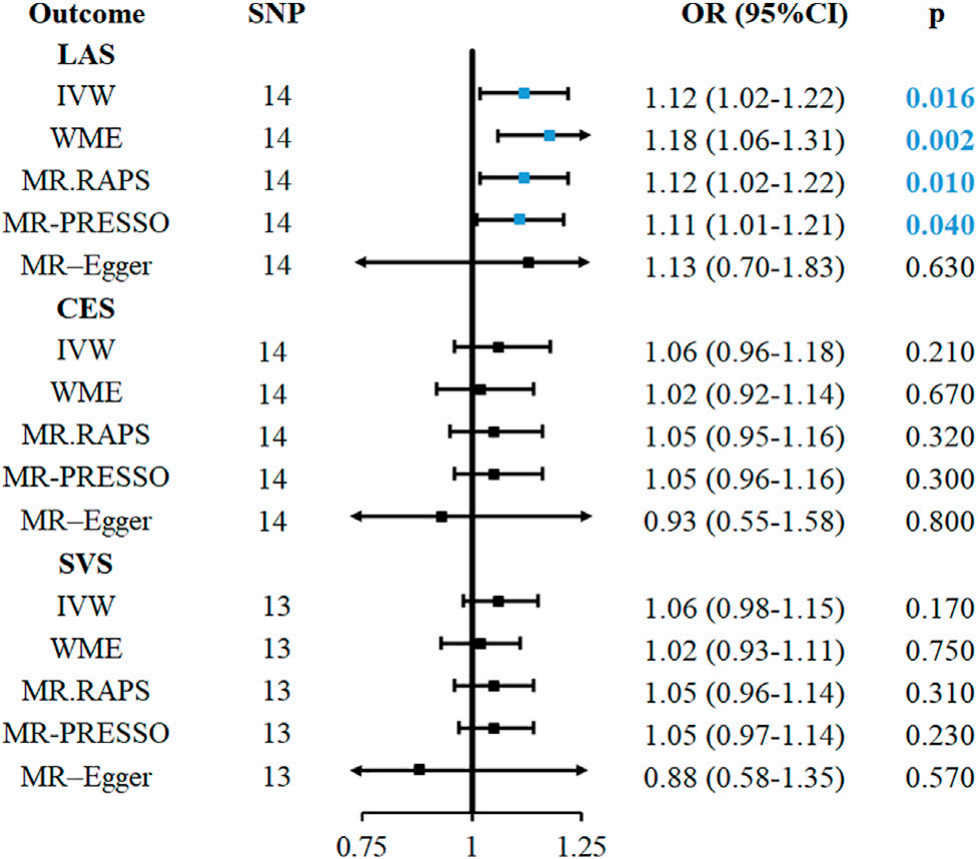
MR analyses causal effect estimates for associations between childhood obesity with IS and its subtypes. CI, confidence interval; IVW, inverse-variance-weighted; MR, Mendelian randomization; OR, odds ratio; SNP, single nucleotide polymorphism; IS, ischemic stroke; LAS, large vessel ischemic stroke; CES, cardioembolic ischemic stroke; SVS, small vessel ischemic stroke; ICH, intracerebral hemorrhage; MR. RAPS, MR-Robust Adjusted Profile Score; MR-PRESSO, MR pleiotropy residual sum and outlier.

**FIGURE 4:**
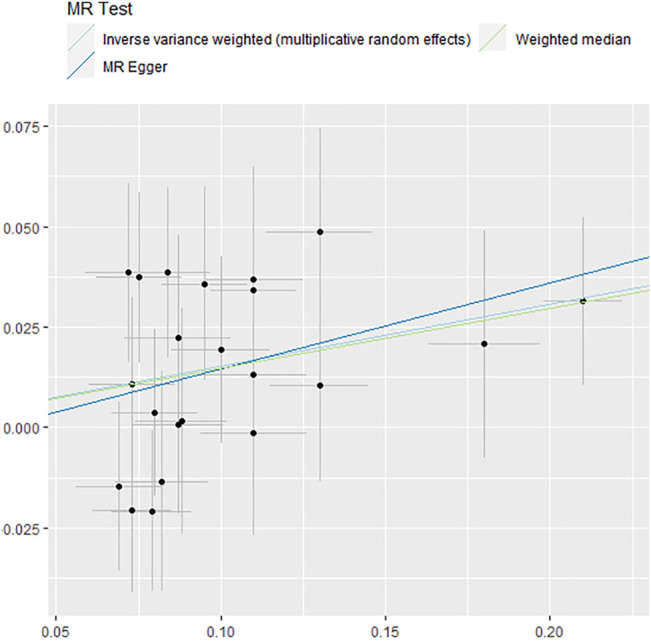
Forest plot of causality between childhood obesity and LAS assessed by MR analysis. MR, Mendelian randomization.

This second analysis was based on the primary analysis of childhood obesity, and we found that it had a causal relationship with stroke and IS. In the IS subtype, childhood obesity was found to be associated with the risk of LAS (OR = 1.12, 95%CI: 1.02–1.22, *p* = 0.016, Figure 3) in the IVW method. In addition, WME (OR = 1.18, 95%CI: 1.06–1.31, *p* = 0.002) and MR-RAPS (OR = 1.12, 95%CI: 1.02–1.22, *p* = 0.01) also provided evidence for this causal relationship (Figure 3). The MR-PRESSO (OR = 1.11, 95%CI: 1.01–1.21, *p* = 0.04) method supported the relationship, but the associations were not significant after Bonferroni correction for multiple comparisons. In the heterogeneity (Q = 20.2, *p* = 0.09), pleiotropy test (*p* = 0.97), no heterogeneity, pleiotropy and outliers were found in childhood obesity-LAS MR analysis. The causal relationship between childhood obesity and LAS is shown in Figure 4, with the slope of each line corresponding to the estimated MR effect in different methods.

Conversely, in childhood obesity-SVS and childhood obesity-CES MR analysis, the causal associations could not be verified in the five analysis methods, as shown in Figure 3. Due to the moderate heterogeneity found in Cochran’s Q test, the random-effect MR model was applied in the analysis of childhood obesity-SVS and childhood obesity-CES. The relationship between childhood-SVS and childhood obesity-CES, which had the same range and direction as the original results, passed the statistical tests after the p-value threshold was adjusted to a genome-wide significant threshold. No evidence of outliers (Supplementary Figures S1D–F) and pleiotropy was confirmed in this analysis.

## Discussion

We performed a two-sample MR analysis to investigate the relationship between childhood obesity and the risk of stroke and its subtypes. The results of our study indicated that childhood obesity can increase the risk of stroke, IS, and LAS, but are not causally related to CES, SVS, and ICH. The sensitivity test provided additional support for a stable and accurate causal outcome. We also considered the reverse causality of cerebrovascular diseases with the risk of childhood obesity, but no significant effect was found.

In previous observational studies, childhood obesity was found to be a common risk factor in cardiovascular and cerebrovascular diseases, cancers, type I diabetes, and many diseases (Lamb et al., 2009; Ajala et al., 2017; Gjærde et al., 2017; Hemani et al., 2018; Weihrauch-Blüher et al., 2019). A cohort study of 307,677 participants, which investigated the incidence of IS with childhood BMI, showed a higher risk of childhood BMI with a high risk of IS at the age ≤55 (Gjærde et al., 2017). Another cohort study indicated that childhood obesity was not associated with the risk of stroke, and was only related to the top 2.5% of the BMI distribution in children (Lawlor and Leon, 2005). Furthermore, obesity in adults may be treated as a potential risk factor for stroke and its subtypes (Marini et al., 2020). To clarify the causal relationship, we utilised GWAS data to confirm the conclusion that childhood obesity can increase the risk of stroke, IS, and LAS.

In addition, given the close relationship between childhood obesity and adult obesity, we analysed whether childhood obesity causes stroke in adults. Current MR studies (Dale et al., 2017; Larsson et al., 2020), which focused on adult obesity and stroke, showed that adult obesity was not associated with stroke, suggesting that childhood and adult obesity may influence stroke in different pathways.

In exploring the potential mechanism among childhood obesity with stroke and its subtypes, we found that childhood obesity can increase arterial stiffness (Tounian et al., 2001) and intima-media thickness (Tounian et al., 2001; Iannuzzi et al., 2004; Ozcetin et al., 2012). It may also lead to metabolic abnormalities and damage the integrity of the vascular endothelium (Celermajer et al., 1992). Endothelial dysfunction then causes subclinical inflammation (Versini et al., 2014) and accelerates the reaction of platelets, neutrophils, and macrophages with the vessel wall (Celermajer et al., 1992). Atherosclerosis then gradually develops. Therefore, childhood obesity plays an important role in the accelerated progression of arterial stiffness and influences the structural and mechanical properties of major vessels (Iannuzzi et al., 2004). Childhood obesity may increase the risk of stroke, IS, and LAS by damaging cerebrovascular vessels through these mechanisms.

Moreover, childhood obesity plays an important role in the development of insulin resistance and abnormal lipid levels (Raitakari et al., 1994). Several studies have shown that insulin resistance is a significant factor in the development of IS via atherosclerosis. Damage to the endothelium and development of foam cells, which is the initial formation of atherosclerosis, can be induced by insulin resistance (Busija and Katakam, 2014; Dorrance et al., 2014). Then, a large number of foam cells will be produced, and vascular smooth muscle cells migrate (Kernan and Inzucchi, 2004; Busija and Katakam, 2014). Lastly, insulin resistance results in vascular damage and thrombosis formation, which contributes to the increased risk of IS (Beckman et al., 2002). In addition, insulin resistance accelerates the adhesion, activation, and aggregation of blood cells, causing hemodynamic disturbances, increasing the risk of IS (Deng et al., 2017). Abnormal lipid levels are another factor of the dependent risk factors of IS and LAS, as they can also affect IS and LAS *via* atherosclerosis. Low-density lipoprotein cholesterol can also increase the risk of IS (OR = 1.12, 95%CI: 1.04–1.20) and LAS (OR = 1.28, 95%CI: 1.10–1.49) (Hindy et al., 2018). Overall, childhood obesity may increase the risk of IS and LAS by hastening the process of atherosclerosis, insulin resistance, and abnormal lipid D.

To the best of our knowledge, our study is the first MR analysis to investigate the causal association between childhood obesity and stroke risk. However, there are limitations in our study. First, only a small number of SNPs were selected as instrumental variables under the rules, so we selected SNPs with a more relaxed value (*p* < 5 × 10^–6^) as suggested by previous studies (Ference et al., 2015; Gao et al., 2019). Another study on BMI in children (Vogelezang et al., 2020) verified the association between BMI and childhood obesity. In addition, we conducted the MR analysis with a p-value less than 5 × 10^–8^, and the causal relationships of childhood obesity-stroke and childhood obesity-IS were consistent with the results in the relaxed condition. Second, the participants included in our study all came from the European ancestry GWAS database, which may not allow the application of the results to other ethnic groups. Therefore, more GWAS and MR analyses in other ethnic groups need to be performed. Third, the features of childhood obesity, such as height and abdominal circumference, were not acquired. These characteristics may be helpful in further classifying childhood obesity. Lastly, the sample size of GWAS studies in ICH is too small, which may limit the power of detecting causal relationships of childhood obesity-ICH in this MR design. However, our study still provided some crucial guidance for the prevention of stroke in the population that had childhood obesity (Bennett and Holmes, 2017).

In summary, our study indicated that childhood obesity has a potential causal association with stroke, IS, and LAS. The prevention of stroke, especially IS and LAS, should be promoted in populations with childhood obesity, and further studies are needed to examine the biological mechanisms underlying this association.

## Data Availability

The original contributions presented in the study are included in the article/Supplementary Material, Further inquiries can be directed to the corresponding author.

## Supplementary Material

Supplementary Figure S1 Leave-one-out Plots for the MR Analyses of childhood obesity with the risk Stroke and ICH, IS, and IS subtypes.
