# Supplementary material for: Childhood Obesity and Risk of Stroke: A Mendelian Randomisation Analysis
Source: Front Genet. 2021 Nov 17;12:727475. doi: 10.3389/fgene.2021.727475 (PMC8638161; doi:10.3389/fgene.2021.727475)
Supplement: Supplementary file 1 [file Table1.docx]

**Supplementary Table 1**

**Supplementary Table 1:** The information of SNP in exposure (childhood obesity) and outcome (stroke and its subtypes).

| SNP | EA | OA | Exposure (childhood obesity ) | | | |  | Outcome (stroke) | | | |  | Outcome (IS) | | | |  | Outcome(ICH) | | | | F |
| --- | --- | --- | --- | --- | --- | --- | --- | --- | --- | --- | --- | --- | --- | --- | --- | --- | --- | --- | --- | --- | --- | --- |
|  |  |  | SE | Beta | Pval | EAF |  | SE | Beta | Pval | EAF |  | SE | Beta | Pval | EAF |  | SE | Beta | Pval | EAF |  |
| rs1040070 | C | G | 0.027 | -0.149 | 2.77E-08 | NA |  | 0.009 | 0.008 | 0.341 | 0.516 |  | 0.009 | 0.009 | 0.328 | 0.509 |  | NA | NA | NA | NA | 30.8 |
| rs10913469 | C | T | 0.033 | 0.177 | 7.99E-08 | NA |  | 0.010 | 0.000 | 0.972 | 0.211 |  | 0.010 | -0.004 | 0.710 | 0.214 |  | 0.823 | 0.004 | 0.067 | 0.948 | 28.9 |
| rs13130484 | T | C | 0.027 | 0.143 | 1.30E-07 | NA |  | 0.008 | 0.011 | 0.166 | 0.396 |  | 0.009 | 0.012 | 0.163 | 0.394 |  | 0.441 | 0.000 | 0.052 | 0.994 | 27.8 |
| rs17697518 | T | C | 0.039 | 0.186 | 1.85E-06 | NA |  | 0.013 | 0.022 | 0.088 | 0.121 |  | 0.014 | 0.025 | 0.072 | 0.119 |  | 0.123 | -0.003 | 0.078 | 0.972 | 22.7 |
| rs256335 | T | C | 0.026 | 0.121 | 3.72E-06 | NA |  | 0.008 | 0.002 | 0.850 | 0.436 |  | 0.009 | 0.002 | 0.850 | 0.431 |  | 0.478 | -0.075 | 0.052 | 0.145 | 21.5 |
| rs28636 | T | C | 0.032 | -0.147 | 3.07E-06 | NA |  | 0.009 | -0.003 | 0.783 | 0.239 |  | 0.010 | -0.002 | 0.819 | 0.245 |  | 0.211 | -0.035 | 0.064 | 0.584 | 21.8 |
| rs4833407 | A | C | 0.027 | 0.123 | 3.88E-06 | NA |  | 0.008 | 0.011 | 0.168 | 0.472 |  | 0.008 | 0.013 | 0.112 | 0.479 |  | 0.420 | -0.002 | 0.052 | 0.974 | 21.4 |
| rs4854344 | T | G | 0.035 | 0.245 | 3.22E-12 | NA |  | 0.011 | 0.011 | 0.310 | 0.832 |  | 0.011 | 0.011 | 0.345 | 0.833 |  | 0.814 | 0.051 | 0.066 | 0.439 | 48.5 |
| rs4864201 | C | T | 0.028 | -0.136 | 1.41E-06 | NA |  | 0.008 | -0.017 | 0.047 | 0.561 |  | 0.009 | -0.014 | 0.113 | 0.552 |  | 0.355 | 0.092 | 0.054 | 0.086 | 23.3 |
| rs571312 | A | C | 0.031 | 0.199 | 1.25E-10 | NA |  | 0.009 | 0.010 | 0.275 | 0.253 |  | 0.010 | 0.014 | 0.150 | 0.254 |  | 0.224 | -0.027 | 0.062 | 0.662 | 41.3 |
| rs6752378 | A | C | 0.026 | 0.170 | 1.05E-10 | NA |  | 0.008 | 0.000 | 0.985 | 0.480 |  | 0.009 | -0.006 | 0.493 | 0.483 |  | 0.453 | -0.054 | 0.052 | 0.297 | 41.9 |
| rs7138803 | A | G | 0.027 | 0.167 | 6.50E-10 | NA |  | 0.008 | 0.005 | 0.529 | 0.364 |  | 0.009 | 0.009 | 0.283 | 0.362 |  | 0.381 | -0.043 | 0.053 | 0.413 | 38.1 |
| rs9299 | T | C | 0.028 | 0.134 | 1.91E-06 | NA |  | 0.008 | -0.017 | 0.043 | 0.611 |  | 0.009 | -0.011 | 0.199 | 0.607 |  | NA | NA | NA | NA | 22.7 |
| rs9568856 | A | G | 0.040 | 0.191 | 1.36E-06 | NA |  | 0.011 | 0.018 | 0.091 | 0.185 |  | 0.011 | 0.015 | 0.177 | 0.190 |  | 0.128 | -0.054 | 0.079 | 0.489 | 23.4 |
| rs9941349 | T | C | 0.027 | 0.198 | 1.16E-13 | NA |  | 0.009 | 0.013 | 0.117 | 0.384 |  | 0.009 | 0.016 | 0.084 | 0.380 |  | 0.414 | -0.042 | 0.052 | 0.416 | 54.9 |

Abbreviation: EA = effect allele, OA = other allele, EAF = effect allele frequency, SNP = single nucleotide polymorphism, IS = ischemic stroke, LAS = large vessel ischemic stroke, CES = cardioembolic ischemic stroke, SVS = small vessel ischemic stroke, ICH = intracerebral hemorrhage.
