# Supplementary figures and images for: Childhood Obesity and Risk of Stroke: A Mendelian Randomisation Analysis
Source: Front Genet. 2021 Nov 17;12:727475. doi: 10.3389/fgene.2021.727475 (PMC8638161; doi:10.3389/fgene.2021.727475)

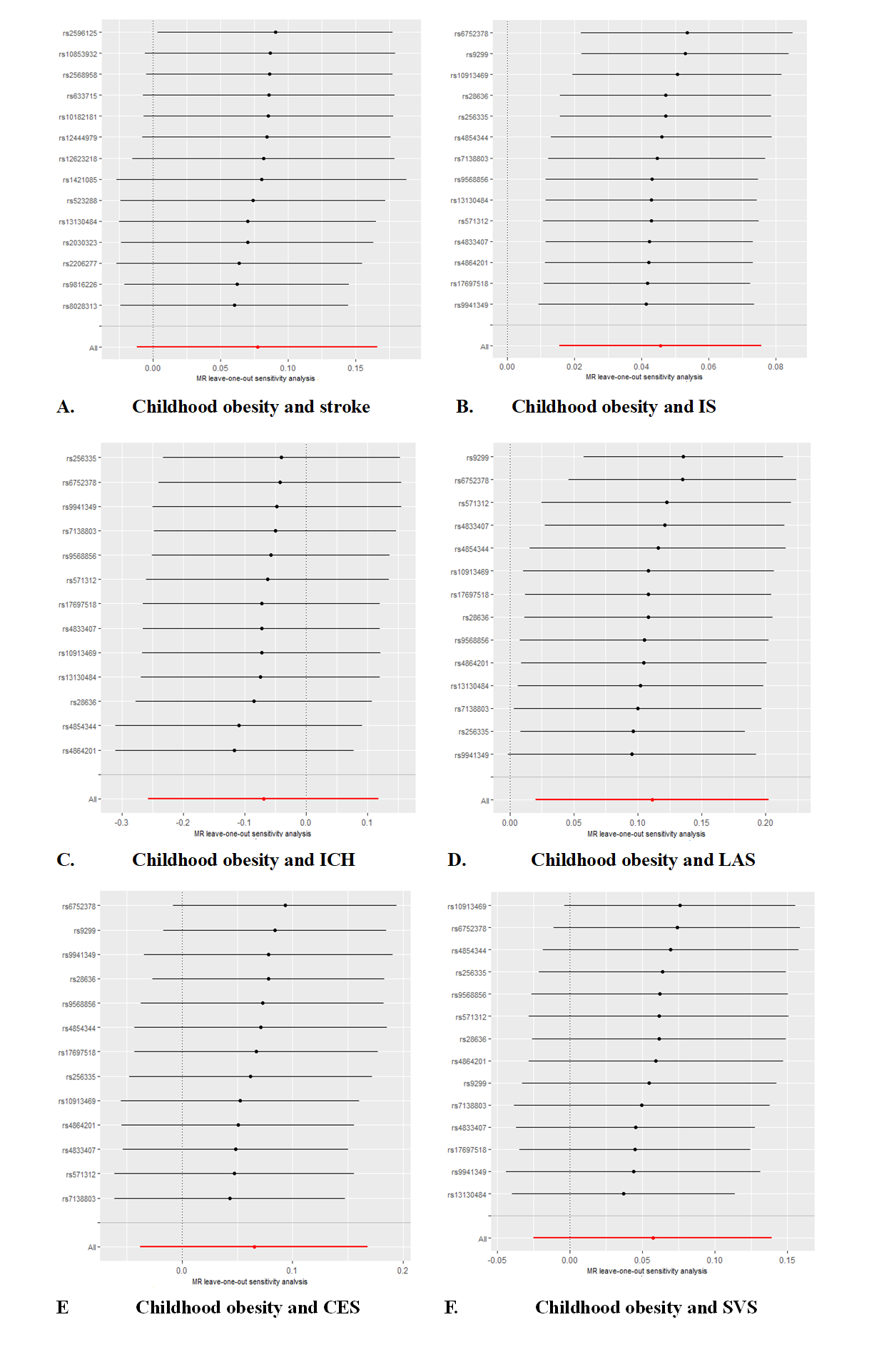

Supplement: Supplementary file 2 [file Image1.tif]
