# Supplementary material for: Childhood Obesity and Risk of Stroke: A Mendelian Randomisation Analysis
Source: Front Genet. 2021 Nov 17;12:727475. doi: 10.3389/fgene.2021.727475 (PMC8638161; doi:10.3389/fgene.2021.727475)
Supplement: Supplementary file 3 [file Table2.docx]

**Supplementary Table 2**

**Supplementary Table 2:** The information of SNP in exposure (childhood obesity) and outcome (IS and IS subtypes).

| SNP | EA | OA | Exposure (childhood obesity ) | | | |  | Outcome (LAS) | | | |  | Outcome(CES) | | | |  | Outcome(SVS) | | | | F |
| --- | --- | --- | --- | --- | --- | --- | --- | --- | --- | --- | --- | --- | --- | --- | --- | --- | --- | --- | --- | --- | --- | --- |
|  |  |  | SE | Beta | Pval | EAF |  | SE | Beta | Pval | EAF |  | SE | Beta | Pval | EAF |  | SE | Beta | Pval | EAF |  |
| rs1040070 | C | G | 0.027 | -0.149 | 2.77E-08 | NA |  | 0.024 | 0.012 | 0.615 | 0.511 |  | 0.019 | 0.023 | 0.232 | 0.533 |  | 0.021 | 0.015 | 0.486 | 0.466 | 30.8 |
| rs10913469 | C | T | 0.033 | 0.177 | 7.99E-08 | NA |  | 0.025 | 0.027 | 0.289 | 0.211 |  | 0.022 | 0.042 | 0.058 | 0.201 |  | 0.021 | -0.033 | 0.110 | 0.224 | 28.9 |
| rs13130484 | T | C | 0.027 | 0.143 | 1.30E-07 | NA |  | 0.021 | 0.034 | 0.097 | 0.393 |  | NA | NA | NA | NA |  | 0.017 | 0.049 | 0.005 | 0.372 | 27.8 |
| rs17697518 | T | C | 0.039 | 0.186 | 1.85E-06 | NA |  | 0.034 | 0.034 | 0.323 | 0.118 |  | 0.028 | 0.005 | 0.859 | 0.124 |  | 0.029 | 0.068 | 0.019 | 0.112 | 22.7 |
| rs256335 | T | C | 0.026 | 0.121 | 3.72E-06 | NA |  | 0.022 | 0.054 | 0.013 | 0.418 |  | 0.018 | 0.015 | 0.389 | 0.451 |  | 0.019 | -0.012 | 0.512 | 0.380 | 21.5 |
| rs28636 | T | C | 0.032 | -0.147 | 3.07E-06 | NA |  | 0.024 | -0.024 | 0.307 | 0.249 |  | 0.021 | 0.026 | 0.235 | 0.228 |  | 0.020 | 0.002 | 0.912 | 0.268 | 21.8 |
| rs4833407 | A | C | 0.027 | 0.123 | 3.88E-06 | NA |  | 0.021 | -0.010 | 0.638 | 0.486 |  | 0.017 | 0.044 | 0.012 | 0.453 |  | 0.017 | 0.035 | 0.037 | 0.520 | 21.4 |
| rs4854344 | T | G | 0.035 | 0.245 | 3.22E-12 | NA |  | 0.027 | 0.018 | 0.503 | 0.838 |  | 0.023 | 0.006 | 0.807 | 0.830 |  | 0.023 | -0.011 | 0.643 | 0.844 | 48.5 |
| rs4864201 | C | T | 0.028 | -0.136 | 1.41E-06 | NA |  | 0.022 | -0.031 | 0.155 | 0.537 |  | 0.018 | -0.039 | 0.029 | 0.589 |  | 0.018 | -0.003 | 0.881 | 0.485 | 23.3 |
| rs571312 | A | C | 0.031 | 0.199 | 1.25E-10 | NA |  | 0.024 | 0.001 | 0.957 | 0.248 |  | 0.020 | 0.044 | 0.030 | 0.251 |  | 0.020 | 0.004 | 0.859 | 0.245 | 41.3 |
| rs6752378 | A | C | 0.026 | 0.170 | 1.05E-10 | NA |  | 0.021 | -0.020 | 0.328 | 0.475 |  | 0.017 | -0.030 | 0.084 | 0.471 |  | 0.017 | -0.016 | 0.338 | 0.482 | 41.9 |
| rs7138803 | A | G | 0.027 | 0.167 | 6.50E-10 | NA |  | 0.021 | 0.039 | 0.069 | 0.364 |  | 0.018 | 0.047 | 0.010 | 0.369 |  | 0.018 | 0.023 | 0.185 | 0.355 | 38.1 |
| rs9299 | T | C | 0.028 | 0.134 | 1.91E-06 | NA |  | 0.021 | -0.041 | 0.056 | 0.617 |  | 0.018 | -0.031 | 0.084 | 0.621 |  | 0.018 | 0.013 | 0.449 | 0.605 | 22.7 |
| rs9568856 | A | G | 0.040 | 0.191 | 1.36E-06 | NA |  | 0.027 | 0.037 | 0.169 | 0.184 |  | 0.024 | -0.007 | 0.758 | 0.175 |  | 0.021 | 0.000 | 0.995 | 0.203 | 23.4 |
| rs9941349 | T | C | 0.027 | 0.198 | 1.16E-13 | NA |  | 0.022 | 0.047 | 0.033 | 0.376 |  | 0.019 | -0.006 | 0.754 | 0.400 |  | 0.018 | 0.033 | 0.072 | 0.344 | 54.9 |

Abbreviation: IVW = inverse-variance-weighted, MR = Mendelian randomization, SNP = single nucleotide polymorphism, HT= heterogeneity test, PT= pleiotropy test, IS= ischemic stroke, LAS= large vessel ischemic stroke, CES= cardioembolic ischemic stroke, SVS=small vessel ischemic stroke, ICH=intracerebral hemorrhage, GT=MR-PRESSO Global Test.
