# Supplementary material for: Childhood Obesity and Risk of Stroke: A Mendelian Randomisation Analysis
Source: Front Genet. 2021 Nov 17;12:727475. doi: 10.3389/fgene.2021.727475 (PMC8638161; doi:10.3389/fgene.2021.727475)
Supplement: Supplementary file 4 [file Table3.docx]

**Supplementary Table 3**

**Supplementary Table 3:** The causal relationship between childhood obesity and stroke in MR analysis (p<5×10^-8^).

| Method | SNP(n) | OR | 95%CI | *p* |
| --- | --- | --- | --- | --- |
| **Stroke** |  |  |  |  |
| MR–Egger regression | 5 | 1.14 | 0.84-1.54 | 0.46 |
| Weighted-median method | 5 | 1.05 | 0.99-1.10 | 0.08 |
| IVW method | 5 | 1.04 | 1.00-1.08 | 0.048 |
| **AIS** |  |  |  |  |
| MR–Egger regression | 5 | 1.16 | 0.85-1.60 | 0.42 |
| Weighted-median method | 5 | 1.06 | 1.00-1.12 | 0.05 |
| IVW method | 5 | 1.05 | 1.00-1.09 | 0.04 |
| **LAS** |  |  |  |  |
| MR–Egger regression | 5 | 1.18 | 0.38-3.66 | 0.80 |
| Weighted-median method | 5 | 1.08 | 0.92-1.25 | 0.34 |
| IVW method | 5 | 1.09 | 0.96-1.25 | 0.18 |
| **CES** |  |  |  |  |
| MR–Egger regression | 5 | 1.01 | 0.26-3.96 | 0.99 |
| Weighted-median method | 5 | 1.01 | 0.89-1.15 | 0.85 |
| IVW method | 5 | 1.06 | 0.90-1.24 | 0.50 |
| **SVS** |  |  |  |  |
| MR–Egger regression | 5 | 0.92 | 0.39-2.16 | 0.85 |
| Weighted-median method | 5 | 1.01 | 0.90-1.14 | 0.85 |
| IVW method | 5 | 1.04 | 0.94-1.15 | 0.48 |
| **ICH** |  |  |  |  |
| MR–Egger regression | 5 | 3.32 | 0.49-22.3 | 0.31 |
| Weighted-median method | 5 | 0.82 | 0.60-1.10 | 0.19 |
| IVW method | 5 | 0.88 | 0.68-1.13 | 0.32 |

Abbreviation: CI = confidence interval; IVW = inverse-variance-weighted; MR = Mendelian randomization; OR = odds ratio; SNP = single nucleotide polymorphism; HT= heterogeneity test; PT= pleiotropy test; AIS= any ischemic stroke; LAS= large vessel ischemic stroke; CES= cardioembolic ischemic stroke; SVS: small vessel ischemic stroke; ICH=intracerebral hemorrhage; REM=random effect model.
